# Chimpanzee intellect: personality, performance and motivation with touchscreen tasks

**DOI:** 10.1098/rsos.170169

**Published:** 2017-05-03

**Authors:** Drew M. Altschul, Emma K. Wallace, Ruth Sonnweber, Masaki Tomonaga, Alexander Weiss

**Affiliations:** 1 Department of Psychology, School of Philosophy, Psychology and Language Sciences, The University of Edinburgh, Edinburgh, UK; 2 Scottish Primate Research Group; 3 Department of Psychology, University of York, York, UK; 4 Department of Cognitive Biology, University of Vienna, Vienna, Austria; 5 Primate Research Institute, Kyoto University, Inuyama, Japan

**Keywords:** personality, animal learning, intelligence, cognitive testing, chimpanzees, primates

## Abstract

Human intellect is characterized by intercorrelated psychological domains, including intelligence, academic performance and personality. Higher openness is associated with higher intelligence and better academic performance, yet high performance among individuals is itself attributable to intelligence, not openness. High conscientiousness individuals, although not necessarily more intelligent, are better performers. Work with other species is not as extensive, yet animals display similar relationships between exploration- and persistence-related personality traits and performance on cognitive tasks. However, previous studies linking cognition and personality have not tracked learning, performance and dropout over time—three crucial elements of cognitive performance. We conducted three participatory experiments with touchscreen cognitive tasks among 19 zoo-housed chimpanzees, whose personalities were assessed 3 years prior to the study. Performance and participation were recorded across experiments. High conscientiousness chimpanzees participated more, dropped out less and performed better, but their performance could be explained by their experience with the task. High openness chimpanzees tended to be more interested, perform better and continue to participate when not rewarded with food. Our results demonstrate that chimpanzees, like humans, possess broad intellectual capacities that are affected by their personalities.

## 1. Introduction

Intellect is highly valued among human societies, and believed to be responsible for advances in all fields of arts and sciences [1]. Intellectual individuals are characterized as intelligent, creative, perceptive, curious, competent, quick to grasp new concepts [2] and strong academic performers [3].

These constituents of human intellect are captured by personality traits [4]. In particular, the Openness and Conscientiousness domains of the human Five-Factor Model [5] are associated with higher achievement [3]; in combination, high scores on these two domains characterize ‘good students’ [5]. The association between Conscientiousness and achievement stems from the fact that individuals high in Conscientiousness possess greater will and motivation to perform, whether it is in the workplace or classroom, despite not necessarily having higher cognitive ability. The association between Openness and achievement is partly explained by the former's moderate correlation with general intelligence (*r* = 0.33) [4].

Openness also overlaps with curiosity [6], need for cognition (individual attraction to tasks that require thinking) [7] and typical intellectual engagement (a mixed construct of personality and intelligence) [8]. These constructs are intercorrelated, and while they are not the same thing as cognitive ability, they are all associated with it [9].

Non-human animal personality, in addition to describing behavioural traits, is associated with cognitive ability [10]. Mice have a general learning ability that is related to exploratory tendencies [11]. Slow-exploring chickadees are more accurate on an instrumental discrimination task, but no quicker to acquire the initial task than fast-explorers [12]. Chimpanzees who explored novel features of and objects in their environment also tended to obtain more rewards from puzzles than less exploratory individuals [13]. Assertive capuchins [14] and friendly macaques [15] were more successful with cognitive tasks, compared with less assertive or friendly conspecifics.

Overall, evidence for an association between personality and cognitive ability in animals has accumulated. Extensive work in great apes, particular chimpanzees, demonstrates that performance can depend on an individual's development, specifically, their experiential history with cognitive tasks [16]. However, researchers lack an analogue for human academic performance. In other species, ‘achievement’ is measured primarily in terms of reproductive fitness, not grade-point average or job performance. Nevertheless, the rudiments of the ‘good student’ are present: chimpanzees that spend more time with puzzles and persist at tool manipulation have greater success in receiving rewards than less persistent individuals [13]. While it is not clear whether the human desire to achieve can be equated with an animal's drive to receive food rewards, animals do possess an intrinsic need for exploration [17]. Clarifying these relationships between personality and performance requires a paradigm in which any individual animal can participate in and depart from the experiment at any time. Learning and dropout among freely participating animals must be tracked and evaluated alongside personality.

Personality traits like those of humans have been found in other primate species [18]. Some of these traits describe individual differences in interest and engagement, but associations with performance on cognitive tasks have been weak. Chimpanzees' interest in a touchscreen task was associated with Openness [19], as was interest in puzzle box tasks [20]. Capuchin monkey participation in two spatial cognition tasks was correlated with Openness, but while performance on the first task was also correlated with Openness, performance on both was negatively correlated with Assertiveness [14]. Rhesus macaques' accuracy on a serial cognition touchscreen task has been associated with Openness and Friendliness [15], but that study could not report on participation. Participation in cognitive tasks appears to be biased by personality [14] and may confound results, e.g. individuals with exploratory tendencies may spend more time around and manipulating an experimental apparatus [13], which may enhance learning simply because these individuals spend more time with the task, rather than because they exhibit greater cognitive ability.

Overall, these earlier findings suggest that intellect's relationship with personality has deep evolutionary roots. To test whether this was the case, we conducted three studies using touchscreen tasks among 19 zoo-housed chimpanzees to determine the degree to which chimpanzee personality domains, particularly Conscientiousness and Openness, are related to engagement and cognitive ability. Personality was measured independently in 2010, 3 years before these studies began. Intellectual engagement was tracked by amount of participation in the tasks; cognitive ability was measured via standard performance metrics for touchscreen tasks: accuracy and response time (RT).

We advance five predictions. First, we expect to replicate previous associations between Openness and greater participation. Second, we would expect individuals (rated as being) high in Conscientiousness to (i) participate for longer periods of time and (ii) show fewer dropouts. Third, we expect that if experience on the cognitive tasks drives the relationships between personality and performance, then the effects of any personality factor would be reduced by controlling for experience on these tasks. Fourth, if performance is not driven by experience, then we would expect that, like in humans, Openness would be associated with better performance. Fifth, we predict that in conditions where food reinforcers are not provided by the task, individuals higher in Openness will still participate.

## 2. Study 1

### 2.1. Methods

Unless otherwise indicated, methods were the same across studies. Participants were a socially housed group of 19 chimpanzees (11 females, 8 males; between 14 and 50 years of age) at the Royal Zoological Society of Scotland's (RZSS) Budongo Trail exhibit at the Edinburgh Zoo. During RZSS pre-specified research blocks, the full group was given simultaneous access to a computer touchscreen set-up in the off-show bedding area of the enclosure. During research times, individuals were free to approach and engage with the apparatus, and could stop participating at any time. Individuals were limited in the number of trials they could complete per day before they were no longer allowed to participate for the rest of that day. Although there were a few cases of individuals stealing rewards from others, this behaviour was rare, and the majority of the time, the chimpanzees took turns interacting with the apparatus without conflict.

Personality was assessed prior to this research, as part of an earlier study [19] by independent researchers and raters. Chimpanzees were rated using the Hominoid Personality Questionnaire (HPQ) [21]^1^ by keepers and researchers who were working at the Budongo Trail exhibit at the time. Chimpanzees were rated by two or three independent raters, all of whom had at least two years of experience with the individuals they rated. The electronic supplementary materials provide full details on personalities, apparatus and enclosure.

Chimpanzees were trained and tested using a two-alternative forced choice task [22]. Participants had to choose one of two visual stimuli presented on the touch-sensitive screen, which required the use of a feature-based or arbitrary associative rule. Each stimulus was composed of a series of square framed, abstract geometrical shapes. Depending on the phase of the study, between two and seven such shapes would be linearly concatenated to form each stimulus. With a few exceptions, the salient shapes of each stimulus were the first and last shapes in each concatenation. All stimuli were procedurally generated and trial unique. During the training phases, all shapes were black, while colour was added for the testing phase.

Participants were randomly selected into one of two groups. Chimpanzees in the first group learned feature-based rules; correct discriminations required that the animal choose the concatenation with the same shaped images at the ends of each concatenation, while ignoring distracting discrepancies, e.g. the colour of different shapes, the length of the concatenations or the incorporation of novel shapes. In the final test, these individuals had to transfer to a new dimension for matching: shape ceased to be salient and the correct choice became the stimulus with matching colours for the last two shapes of the concatenation. Chimpanzees in the second group learned associative rules. Having first learned to associate five pairs of shapes, these chimpanzees also needed to choose the stimulus with the first shape of a pair in the first position of the concatenation, and the second of the pair in the last position, while ignoring distractions such as mismatched colour, incorrect positioning or inverted pairs.

Each training or testing session consisted of 12 trials, and within one daily research block, an individual could engage in up to four sessions. During training sessions, if a chimpanzee chose the correct stimulus, they received acoustic reinforcement, a ‘clicker’ sound familiar to the chimpanzees from husbandry training, and a food reward, then the task would advance to the next trial. Food rewards varied depending on the preferences of the individuals and availability during any given day, but rewards were chosen so as to provide maximal incentive to the chimpanzee using the apparatus. If the chimpanzee chose incorrectly, an unappealing, irregular series of sounds was played, and a time-out penalty screen was displayed for 3 s. The same trial would then be repeated until the individual chose the correct stimulus.

To proceed^1^ to the next stage of training, an individual had to correctly complete 33 of 48 consecutive trials. When an individual reached the testing phase, half of the trials would be stimuli pairings from earlier training stages, and the other half would be novel stimuli pairings: test stimuli that were neither fed back nor rewarded. Depending on which of two experimental groups the individuals were assigned to, the chimpanzees would have access to at most seven or 10 different tests within the testing phase. Each test consisted of a fixed number of trials—between 30 and 60. Although chimpanzees were encouraged to continue working through training and testing, they could stop participating at any time during the experiments (see Sonnweber *et al*. [22] for full details of all experimental conditions and stimuli).

### 2.2. Results

**Figure 1.**
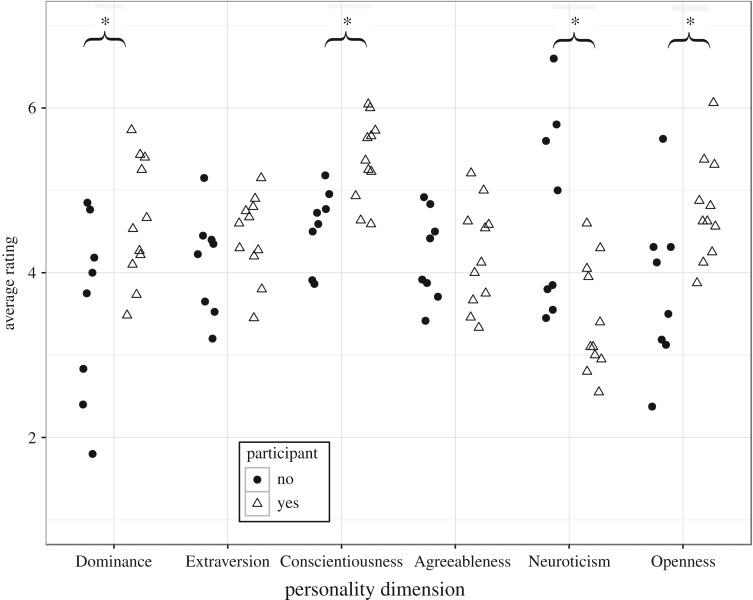
Filled black circles represent non-participants, hollow triangles represent participants. Non-participants did not engage with the tasks at all, while participants did to varying degrees; an analysis of dropout among these individuals can be found in table 1. Asterisks represent statistically significant differences between the groups.

To assess differences in participation, we first compared the personalities of 11 individuals who participated and eight individuals who did not participate (figure 1). To be considered a participant, a chimpanzee must have completed at least a session worth of trials in one sitting. The difference between participants and non-participants was clear, as the chimpanzees who did participate all completed between 224 and 3829 trials.

**Table 1.** Regression analyses from Study 1. Bold text indicates significant variables, where confidence intervals do not overlap with 0.

	dropout from study		accuracy	
parameter	*β*	95% CI	*β*	95% CI
Dominance	1.12	[−1.38, 3.62]	−0.15	[−0.48, 0.15]
Conscientiousness	**−2.80**	**[−4.50, −1.11]**	0.25	[−0.45, 0.52]
Openness	−0.98	[−2.47, 0.50]	0.13	[−0.03, 0.41]
Neuroticism	1.66	[−0.98, 4.29]	0.04	[−0.34, 0.42]
Agreeableness	**2.21**	**[0.83, 3.59]**	−0.06	[−0.29, 0.18]
Extraversion	−1.00	[−2.21, 0.21]	**0.22**	**[0.01, 0.43]**
Date	—	—	**0.30**	**[0.22, 0.38]**

Participating individuals were (rated as being) higher in Dominance (*t* = 2.31, d.f. = 11.44, *d* = 1.14, *p* = 0.04), Conscientiousness (*t* = 3.61, d.f. = 15.84, *d* = 1.67, *p* = 0.002) and Openness (*t* = 2.39, d.f. = 10.98, *d* = 1.19, *p* = 0.04), and lower in Neuroticism (*t* = −2.69, d.f. = 10.22, *d* = 1.36, *p* = 0.02). These differences survived Benjamini–Hochberg correction for multiple tests.

Of the 11 participating chimpanzees, individuals showed differing amounts of participation. Some chimpanzees stayed with the tasks longer than others, e.g. six chimpanzees progressed to the testing phase and only three completed testing. To examine the effect of personality on dropout over the course of training and testing, we fitted a Cox proportional hazards regression model with Gaussian frailty effects to the training and testing data (table 1). Chimpanzees higher in Conscientiousness were 16 times less likely to drop out; chimpanzees higher in Agreeableness were nine times more likely to drop out.

We then modelled associations between personality and learning speed using another Cox model to predict the total number of stages completed and a Poisson mixed model to predict the number of trials it took to reach criterion during training stages (electronic supplementary material, table S3). None of the personality predictors were consistently related to learning speed.

Accuracy across the task was generally good (*M* = 61%). Although individuals displayed overall accuracy as high as 69%, two participants did not perform above chance (the lowest average accuracy was 46%). Accuracy across the training and testing stages was analysed with generalized linear mixed models (GLMM). When personality alone was used to predict trial accuracy, Extraversion and Conscientiousness were positively associated with accuracy. However, when date (representing experience with the task) was added as a predictor, only Extraversion remained significant (table 1).

RTs in all studies were calculated as the time difference between stimulus onset and the chimpanzee's first touch response to the screen, which initiated a visual and sometimes auditory stimulus change. GLMMs of RTs per trial revealed associations between faster RTs and higher Conscientiousness (*β* = −0.53, 95% CI [−0.92, −0.14]), Openness (*β* = −0.38, 95% CI [−0.76, −0.01]) and Extraversion (*β* = −0.39, 95% CI [−0.67, −0.10]) (electronic supplementary material, table S5). Chimpanzees can be sloppy performers, on a trial-by-trial basis, and so we tracked how many touches to the screen it took for an individual to select its intended target. A GLMM of the number of touches per trial indicated that higher Conscientiousness (*β* = −0.38, 95% CI [−0.76, −0.00]) was associated with fewer touches (electronic supplementary material, table S6).

## 3. Study 2

### 3.1. Methods

The chimpanzees had free access to the experimental apparatus in the research areas, or pods, of the enclosure. Unlike the bedding area where Study 1 was conducted, the research pods were viewable to the public. Otherwise, the procedure was very similar; during research times, individuals were free to approach and engage with the apparatus, and could stop participating at any time. After completing a pre-specified number of trials, an individual would no longer be allowed to participate in the task.

Study 2 used a delayed match-to-sample (DMTS) task (electronic supplementary material, figure S1): participants were shown a start stimulus which had to be touched to continue, after a 0.5 s delay a sample image was displayed in a randomly assigned location on a 3 × 3 grid. The sample also needed to be touched, and after another 0.5 s delay, two images, the sample, which again had to be chosen, and a distractor, were presented on the 3 × 3 grid. All samples and distractors were selected randomly from a large bank of colour photographic images. If a chimpanzee chose correctly, they received acoustic reinforcement and a food reward. If the chimpanzee chose incorrectly, an unappealing acoustic signal was played, and a time-out penalty screen was displayed for 2 s. After correct and incorrect trials, there was a 0.5 s intertrial interval, and no repetition of trials, i.e. no correction procedure was used to amend incorrect responses by the chimpanzees.

We also collected ordinal data on the chimpanzees' daily engagement in the research areas. Every day, individuals were each assigned to one of three escalating levels: 0—the individual did not enter the research area or did not show any interest in the touchscreen, 1—the individual showed interest in and approached the touchscreen, but did not complete any trials and 2—the individual interacted with the touchscreen and completed as least one trial.

### 3.2. Results

Engagement data were analysed with cumulative link mixed models (CLMM). These models indicated that high Openness and low Agreeableness were associated with increased engagement with the DMTS task (table 2).

**Table 2.** Regression analyses from Study 2. Bold text indicates significant variables, where confidence intervals do not overlap with 0.

	engagement		accuracy	
parameter	*β*	95% CI	*β*	95% CI
Dominance	−0.71	[−1.74, 0.33]	0.20	[−0.07, 0.47]
Conscientiousness	0.34	[−0.71, 1.40]	0.09	[−0.14, 0.32]
Openness	**0.77**	**[0.19, 1.36]**	**0.22**	**[0.01, 0.43]**
Neuroticism	−0.73	[−1.71, 0.25]	0.00	[−0.21, 0.21]
Agreeableness	**−1.11**	**[−2.00, −0.22]**	−0.10	[−0.33, 0.13]
Extraversion	0.43	[−0.51, 1.37]	0.01	[−0.16, 0.18]
Location	**−1.27**	**[−1.76, −0.82]**	—	—

The DMTS task was slightly more difficult for the chimpanzees then the two-choice forced alternative task in Study 1. Individuals displayed overall accuracy as high as 67%, but again two participants did not perform above chance (the lowest average accuracy was 49%; *M* = 54%). The association between personality traits and performance was modelled with GLMMs, using the same approach as in Study 1. Accuracy on the DMTS task was only associated with higher Openness (table 2). Date was omitted from the final model because including it did not improve model fit (*χ*^2^ = 1.60, d.f. = 1, *p* = 0.21). Analyses of RTs revealed consistent associations (electronic supplementary material, table S8) between faster responses, to the choice stimulus and at the test screen, and higher Extraversion (*β* = −0.34, 95% CI [−0.63, −0.05]; *β* = −0.39, 95% CI [−0.55, −0.23]). Quicker RTs at the test screen were also associated with lower Dominance (*β* = 0.49, 95% CI [0.24, 0.74]), higher Neuroticism (*β* = −0.48, 95% CI [−0.77, −0.20]) and higher Agreeableness (*β* = −0.25, 95% CI [−0.44, −0.06]), though the power to detect Agreeableness in this instance was quite low (26%), suggesting that this result is a false positive.

## 4. Study 3

### 4.1. Methods

Study 3 was divided into six phases. First, participants were trained on a new touchscreen task, which consisted of three horizontal bar buttons, which could appear in three positions (electronic supplementary material, figure S4). The buttons were defined by their pattern and the musical sounds they played when pressed. The positions of the buttons were randomized on each trial, though every position was filled and not every button appeared on every trial. The chimpanzees were introduced to every button individually and then in combination with all others over the course of the first four phases. In the fifth phase, the layout was changed to a 3 × 3 grid (electronic supplementary material, figure S5) similar to Study 2. During training, pressing any button would result in the participant being rewarded with a piece of grape, so there were no wrong answers. The only criterion to advance was that individuals needed to complete 10 trials of phases 1 through 4, and 40 trials of phase 5.

The sixth phase represented a shift in procedure. After training the chimpanzees for 12 days on phases 1 through 5, training rewards were removed, and the chimpanzees were allowed to interact with the apparatus. After all of the trained chimpanzees experienced the unrewarded version of the task, 11 days of testing began. Each day the pods were baited with pellets and straw, and the experimental programme was made available. The experimental programme did not differ from previous phases: a 3 × 3 of grid of buttons was displayed, sound would be played when a chimpanzee pressed a button, the screen would be randomly redrawn with the button in different locations, and no rewards were given out by the apparatus.

We monitored the time chimpanzees spent in the pods and engaged with the screen. As in Study 2, chimpanzees were free to engage with the same apparatus in the indoor research pods at any point during the research times, during all phases. All chimpanzees could participate, regardless of whether they had previously participated, and how many phases they might have completed.

### 4.2. Results

Seven chimpanzees participated in the five training phases. The differences in personality between the chimpanzees who did and did not complete training are shown in electronic supplementary material, figure S5. Tests of Conscientiousness (*t* = 2.285, *d* = 1.05, *p* = 0.04), Neuroticism (*t* = −1.487, *d* = −0.73, *p *= 0.16) and Openness (*t* = 2.295, *d* = 1.11, *p* = 0.04) revealed that the trained group was higher in Conscientiousness and Openness, but the results were not significant after Benjamini–Hochberg correction.

The results of our analyses of engagement are shown in table 3. We first regressed personality onto the amount of time that each chimpanzee spent in the pods over the course of every research block. A negative binomial GLMM indicated that chimpanzees rated higher in Extraversion spent more time in the pods (*β* = 0.78, 95% CI [0.06, 1.51]) during these blocks. All chimpanzees spent a majority of their time foraging for pellets, collecting straw and grooming, so to assess their interest in the touchscreen we regressed personality onto the number of approaches to the touchscreen, again using a negative binomial model. Chimpanzees higher in Conscientiousness (*β* = 1.09, 95% CI [0.15, 2.16]) and lower in Agreeableness (*β* = −0.93, 95% CI [−1.63, −0.28]) made more approaches to the screen. Finally, we regressed personality onto the amount of time the chimpanzees spent physically engaged with the screen using a Poisson GLMM. Chimpanzees higher in Openness (*β* = 0.52, 95% CI [0.21, 0.83]) and lower in Extraversion (*β* = −0.36, 95% CI [−0.66, −0.05]) spent more time engaged with the screen, despite not being rewarded with food.

**Table 3.** Regression analyses of engagement data from Study 3. Bold text indicates significant variables, where confidence intervals do not overlap with 0.

	time spent in pods		approaches to screen		time spent at screen	
parameter	*β*	95% CI	*β*	95% CI	*β*	95% CI
Dominance	−0.14	[−1.60, 1.31]	−0.19	[−1.67, 1.15]	0.26	[−0.20, 0.73]
Conscientiousness	−0.25	[−1.13, 0.63]	**1.09**	**[0.15, 2.16]**	0.21	[−0.17, 0.59]
Openness	−0.06	[−0.79, 0.66]	0.46	[−0.43, 1.46]	**0.52**	**[0.21, 0.83]**
Neuroticism	−0.61	[−2.08, 0.85]	0.10	[−1.43, 1.48]	0.41	[−0.10, 0.91]
Agreeableness	−0.28	[−0.96, 0.42]	**−0.93**	**[−1.63, −0.28]**	0.19	[−0.04, 0.43]
Extraversion	**0.78**	**[0.06, 1.51]**	0.69	[−0.19, 1.70]	**−0.36**	**[−0.66, −0.05]**

## 5. Power analyses

Where appropriate and feasible, we carried out power analysis simulations on our reported regression models to determine the power of the significant effects of personality that we found. The results of these analyses are shown in table 4. Mean power is reported instead of median power because the mean was more conservative. The mean power of all our results fell between 67% and 89%, indicating adequate to good power [23].

**Table 4.** Power analyses for regression models across studies (*n*, number of significant effects for which power could be calculated).

parameter	mean power	range	*n*
Dominance	0.79	0.63–0.95	4
Conscientiousness	0.83	0.65–0.93	7
Openness	0.76	0.50–0.96	4
Neuroticism	0.77	—	1
Agreeableness	0.67	0.26–0.95	4
Extraversion	0.89	0.81–0.97	10

## 6. Discussion

These studies suggest that chimpanzees, like humans, possess intellectual capacities (e.g. engagement, curiosity) and non-intellectual capacities (e.g. reward seeking, precision in touch responses) that are tied to different aspects of personality and performance. Chimpanzees higher in Conscientiousness were more likely to participate; however, when rewards were removed they abandoned the task. These chimpanzees would frequently approach the apparatus, presumably to check if rewards had been reinstated, but in spite of this, they did not spend more time in front of the screen than individuals lower in Conscientiousness. Chimpanzees higher in Conscientiousness were also less likely to drop out, but when we controlled for the effects of training, Conscientiousness did not predict accuracy. The positive relationship between accuracy and Conscientiousness was the only association we found that was eliminated by controlling for training, suggesting that high Conscientiousness chimpanzees, much like high Conscientiousness humans [3], are not inherently smarter, but achieve high levels of performance through greater expertise.

Agreeableness was consistently associated with lower participation rates and higher dropout rates. Low Agreeableness, probably less altruistic [21], chimpanzees were often inclined to spend time interacting with the touchscreen, monopolizing rewards from the task, and preventing others from participating.

While several models indicated that high Extraversion was associated with higher accuracy and higher participation, these findings were largely inconsistent; for example, chimpanzees rated higher in Extraversion showed significantly less interest in the task in Study 3. This inconsistency in associations between Extraversion and engagement is reminiscent of findings in humans [24]; Extraversion is modestly correlated with intelligence (*r* = 0.08), but not associated with academic performance. On the other hand, high Extraversion was consistently associated with faster RTs, which is consistent with the view that differences in Extraversion are underlain by differences in motor mechanisms [25].

Neither Dominance nor Neuroticism displayed any major or consistent contributions to performance or participation. This is surprising considering the importance of social hierarchy to chimpanzee behaviour [26]. Earlier evidence in other species, notably macaques, suggested that rank characteristics affected individual rhesus macaques' expression of what they learned, but only in mixed social contexts [27]. However, more recent work found that low rank predicted higher training success in long-tailed macaques, but this effect was not as influential as that of personality dimensions that were not significantly correlated with rank [28]. There is thus little evidence for a consistent relationship between Dominance or similar personality dimensions (e.g. Confidence or Assertiveness) and non-social cognition.

Previous research with these chimpanzees showed that individuals who were higher in Neuroticism were more vigilant and engaged in more self-directed behaviours while participating in cognitive research [19]. Test anxiety, known to negatively impact performance on intelligence tests, is more common in high Neuroticism humans [4]. Despite showing signs of anxiety during testing, high Neuroticism chimpanzees did not perform more poorly than other chimpanzees. Having learned the importance of test taking over a lifetime [29], the test anxiety effect may reflect a tendency in humans to assign greater meaning to testing outcomes.

Openness was repeatedly associated with performance and participation. Most tellingly, chimpanzees high in Openness remained interested even when they were no longer rewarded, despite the fact that this took time away from opportunities to forage for free rewards. Openness was not associated with every measure of performance, however. Thus, while Openness partly overlaps with cognitive ability, Openness is also related to higher participation and curiosity about, and interest in, something intrinsic to the tasks themselves. These associations position chimpanzee Openness, like human intellect [2], close to a need for cognition.

Our findings are similar to what has been demonstrated in humans, particularly the connections between Conscientiousness and achievement [3], and Openness and need for cognition [9]. Nevertheless, these studies were conducted with only a single group of chimpanzees. Future studies should be conducted in different, large groups. Moreover, the evidence on the covariance of personality and performance has been disproportionately focused on chimpanzees. The attributes shared between human and chimpanzee intellect suggest that the roots of human achievement, intelligence and personality run far deeper than our own taxonomic family. To understand how far back these commonalities stretch, we need to study personality in concert with engagement and performance in other intelligent species.

## Supplementary Material

Supplemental methods and results

## Footnotes

1 The HPQ is available online at http://extras.springer.com/2011/978-1-4614-0175-9/weiss_chimpanzee_personality.pdf.

## Data accessibility

The datasets supporting this article are available on the Dryad Digital Repository at http://dx.doi.org/10.5061/dryad.2v075 [30].
