# Supplementary material for: Chimpanzee intellect: personality, performance and motivation with touchscreen tasks
Source: R Soc Open Sci. 2017 May 3;4(5):170169. doi: 10.1098/rsos.170169 (PMC5451826; doi:10.1098/rsos.170169)
Supplement: Supplemental methods and results [file rsos170169supp1.docx]

**Chimpanzee Intellect: Personality, Performance, and Motivation with Touchscreen Tasks: Supplemental Materials**

Drew M Altschul^1,2^, Emma K Wallace^2,3^, Ruth Sonnweber^4^, Masaki Tomonaga^5^, & Alexander Weiss^1,2^

1. The University of Edinburgh, Department of Psychology
2. Scottish Primate Research Group
3. University of York, Department of Psychology
4. University of Vienna, Department of Cognitive Biology
5. Primate Research Institute, Kyoto University

**Supplemental Materials**

***Methods***

*Living Enclosure*

The Royal Zoological Society of Scotland’s Edinburgh Zoo Budongo Trail exhibit is a space purpose-built for chimpanzee living and research. The exhibit consists of an off-exhibit area (21.45 m^2^), access tunnels (34.6 m), three indoor enclosures (125 m^2^ and 14 m high), an outdoor enclosure (1,832 m^2^) with a research hut attached via windows, and an multi-chamber indoor research area (26.5 m^2^), referred to as the research pods [1]. The enclosure was designed to both facilitate research and allow the chimpanzees to split into multiple sub-groups, i.e. express natural fission-fusion behaviour. A schematic of the enclosure is presented as Figure S1.


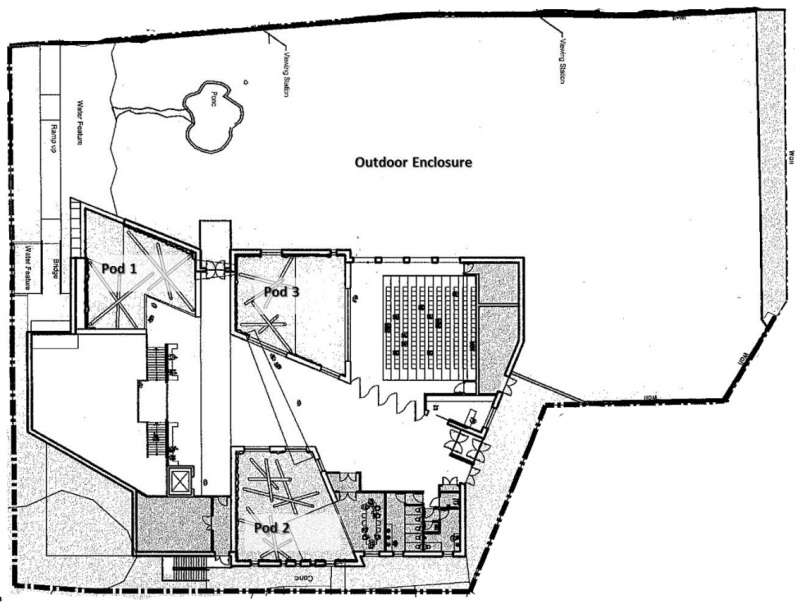


**Figure S1.** Layout of Budongo Trial enclosure.

Water was available ad libitum. The chimpanzees were fed via scatter feeding at least four times a day.

*Participant Demographics*

The study group consisted of 19 chimpanzees (eleven females, eight males; between 14 and 50 years of age). One chimpanzee died between the end of study 1 and the start of study 2. Ethical approval was obtained from the University of Edinburgh Biological Services Ethical Review Committee, and the Budongo Trail Scientific Committee.

*Personality Assessment*

In 2010, all chimpanzees were rated by between two and four zoo caretakers using the 54-item Hominoid Personality Questionnaire, an instrument composed of adjective items, each accompanied by two or three sentences of explanation and a 1 – 7 Likert scale [2]. Raters were instructed to rate individuals using the full spectrum of the scale, and not discuss their ratings with others. Intraclass correlations (ICCs)[3] were calculated for all items, and are showing in Table 1. Items with ICCs below zero were excluded from further analysis, and aggregate personality scores were calculated without them. In this manner, ‘impulsive’, ‘predictable’, and ‘clumsy’ were removed.

| **Table S1.** Intraclass correlations of personality items | | | |
| --- | --- | --- | --- |
| Item | ICC(3,1) | ICC(3,k) | Personality Loading^a^ |
| Fearful | 0.530 | 0.772 | -D |
| Dominant | 0.822 | 0.933 | +D |
| Persistent | 0.242 | 0.489 | +D |
| Cautious | 0.220 | 0.458 | -D |
| Stable | 0.270 | 0.526 | -N |
| Autistic | 0.699 | 0.874 | +N |
| Curious | 0.313 | 0.577 | +O |
| Thoughtless | 0.126 | 0.301 | -C |
| Stingy | 0.440 | 0.702 | +D |
| Jealous | 0.232 | 0.476 | -C |
| Individualistic | 0.031 | 0.087 | -E |
| Reckless | 0.464 | 0.722 | -C |
| Social | 0.320 | 0.585 | +E |
| Distractible | 0.500 | 0.750 | -C |
| Timid | 0.708 | 0.879 | -D |
| Sympathetic | 0.439 | 0.701 | +A |
| Playful | 0.477 | 0.732 | +E |
| Solitary | 0.766 | 0.907 | -E |
| Vulnerable | 0.350 | 0.618 | -D |
| Innovative | 0.566 | 0.797 | +O |
| Active | 0.660 | 0.853 | +E |
| Helpful | 0.188 | 0.409 | +A |
| Bullying | 0.650 | 0.848 | +D |
| Aggressive | 0.726 | 0.888 | -C |
| Manipulative | 0.392 | 0.659 | +D |
| Gentle | 0.623 | 0.832 | +A |
| Affectionate | 0.199 | 0.427 | +E |
| Excitable | 0.331 | 0.597 | +N |
| Impulsive | -0.051 | -0.172 | -C |
| Inquisitive | 0.456 | 0.716 | +O |
| Submissive | 0.780 | 0.914 | -D |
| Cool | 0.381 | 0.649 | -N |
| Dependent | 0.495 | 0.746 | -D |
| Irritable | 0.218 | 0.455 | -C |
| Unperceptive | 0.373 | 0.641 | -C |
| Predictable | -0.011 | -0.035 | +C |
| Decisive | 0.470 | 0.727 | +D |
| Depressed | 0.442 | 0.703 | -E |
| Conventional | 0.170 | 0.381 | +A |
| Sensitive | 0.017 | 0.051 | +A |
| Defiant | 0.533 | 0.774 | -C |
| Intelligent | 0.489 | 0.742 | +O |
| Protective | 0.619 | 0.830 | +A |
| Quitting | 0.504 | 0.753 | -C |
| Inventive | 0.626 | 0.834 | +O |
| Clumsy | -0.012 | -0.036 | -C |
| Erratic | 0.438 | 0.700 | -C |
| Friendly | 0.062 | 0.165 | +E |
| Anxious | 0.633 | 0.838 | -D |
| Lazy | 0.428 | 0.692 | -E |
| Disorganized | 0.313 | 0.578 | -C |
| Unemotional | 0.496 | 0.747 | -N |
| Imitative | 0.470 | 0.727 | +E |
| Independent | 0.519 | 0.764 | +D |
|  |  |  |  |

^a^ D, Dominance; C, Conscientiousness; O, Openness; N, Neuroticism; A, Agreeableness; E, Extraversion.

*Prior Experience*

All chimpanzees had been habituated to the research facilities. 14 chimpanzees had received some training on a two-alternative forced choice (2AFC) task. 11 participated on a regular basis [2].

*Apparatus & Access*

In study 1, the apparatus consisted of a 15” touch-screen, an Apple Mac Mini, a second monitor for the experimenter, a keyboard, optical mouse, and speakers. This equipment was all mounted on a rolling table so that the apparatus could be moved around the off-exhibit areas, to where the chimpanzees could interact with it.

In study 2, the apparatus was mounted to a research window, opening onto either the indoor research pods, or the outdoor enclosure, depending on the day. In study 3, the apparatus was only used indoors. In both studies, the apparatus consisted of a 17” touchscreen monitor, a customized PC running Linux Mint, a monitor for the experimenter, a keyboard, optical mouse, speakers, and Bio-Medica Ltd. Universal Feeder [4].

All studies were participatory; the chimpanzees were free to come and go from the research areas at any time during the research sessions. When reinforcement was in place for correct trials, individuals would be given a food reward for every correct trial (e.g. half of a grape).

*Experimental Programs*

All programs were written in Python, and studies 2 and 3 used additional Kivy libraries. The details of the experimental approach of study 1 can be found in Sonnweber et al. [5]. Figure S2 shows the procedure for a sample trial in study 2.

Stimuli in study 1 were generated ad-hoc by the experimental program. Stimuli in study 2 were gathered in advance. Stimulus banks were sufficiently large and disparate that they could not be memorized, so all stimuli were treated as trial unique. Stimuli in study 3 did not change, as rewards were not contingent on any stimulus attributes.


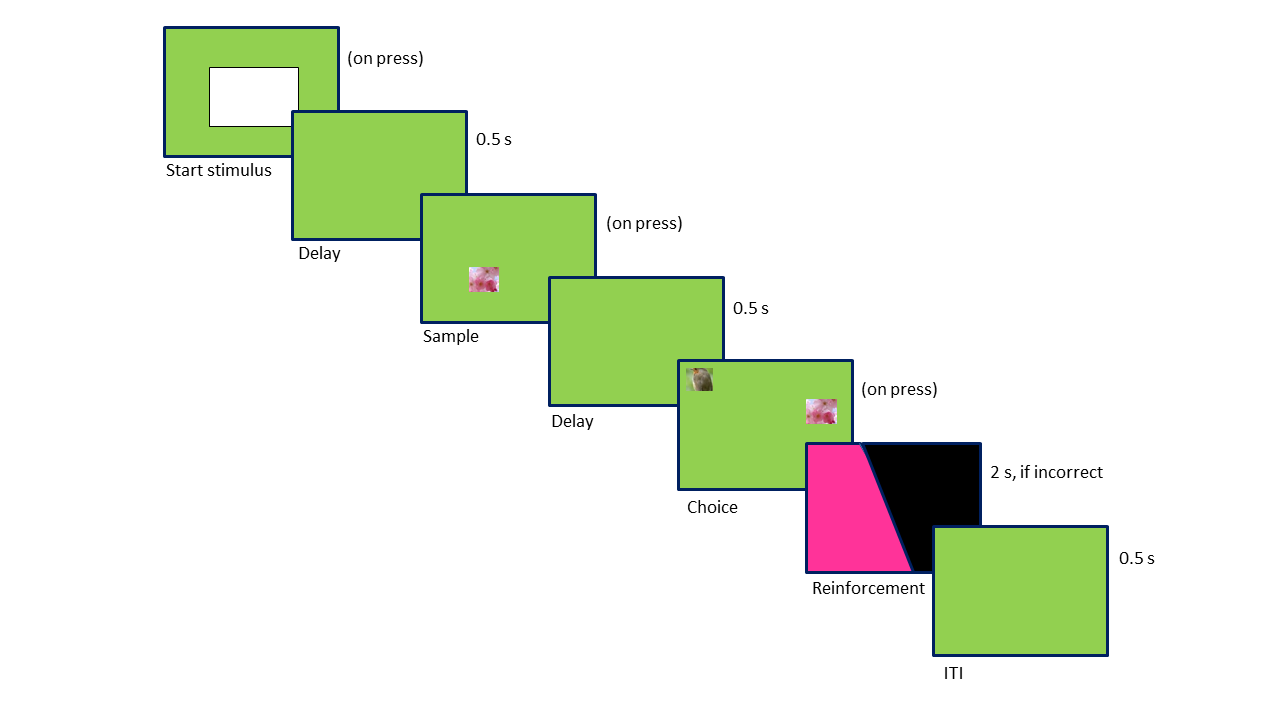


**Figure S2.** Procedure for task used in Study 2.

*Engagement*

In study 2, we collected ordinal data intended to quantify the chimpanzees’ differential levels of interest in the touchscreen task, on a daily basis. No presence in the research area at any time during a research slot was coded as 0. Presence without interaction with the apparatus was coded as 1. Presence and interaction was coded as 2.

In study 3, we kept track of when individual chimpanzees progressed through different stages of training. During testing, we used audio and video recorders to keep track of when every chimpanzee entered and exited the research pods, as well as when they approached and withdrew from the touchscreen apparatus.

**Results**

All data analyses used standardized, centred variables, following the recommendations of Gelman [6]. To make predictors comparable in regression models, continuous variables were centred and divided by 2 standard deviations, and dichotomous variables were centred.

***Study 1***

*Interest in participating*

Two-sample Welch’s *t*-tests were conducted, informed by Figure 1 (main text). Accordingly, only Dominance, Conscientiousness, Openness, and Neuroticism were tested, as the other personality dimensions showed no differences at the group level.

*Drop-out*

There were two training tracks for participating chimpanzees, and the two tracks had different numbers of stages during training and testing. Our data thus treated each stage for every chimpanzee as a different entry; the number of trials the chimpanzee remains at the stage was recorded, and the stage could either end with a drop-out event, or the chimpanzee continued to the next stage, in which case the entry would be censored. Gaussian frailty effects were included on an individual basis, since each individual had multiple entries, and for each distinct stage.

In addition to the Cox model, we fitted an accelerated failure time (AFT) model to these data, as Cox and AFT models are comparable, but have different advantages. The model specification was the same, except that due to technical limitations, frailty effects were only included for the individual. Based on likelihood-ratio tests, the Weibull distribution was the best distribution for the AFT model given these data. Compared to the Cox model, however, the AFT model did not improve fit (likelihood-ratio test; *χ* = -76.2, df = 0.06, *p* ≈ 1). Nevertheless, the results of both models were not substantively different, and while power could not be calculated for the AFT model, the power to detect the significant effects of Conscientiousness and Agreeableness in the Cox model were 93% and 95%, respectively. The AFT model is described in Table S2.

| **Table S2.** AFT model of drop-out from study 1 | | |  |
| --- | --- | --- | --- |
| Parameter | *β* | 95% | C.I. |
| Dominance | -1.05 | [-3.77, | 1.67] |
| Conscientiousness | **2.96** | **[1.04,** | **4.87]** |
| Openness | 1.08 | [-0.55, | 2.71] |
| Neuroticism | -1.29 | [-4.35, | 1.78] |
| Agreeableness | **-2.45** | **[-3.69,** | **-1.20]** |
| Extraversion | 1.07 | [-0.29, | 2.42] |
|  |  |  |  |
| scale | 0.546 |  |  |

*Learning Speed*

Learning speed was quantified in two ways. First, we fitted another Cox model, using the same number of trial data from our earlier drop-out model, but if an individual reached criterion after that many trials they were assigned a completion event, otherwise they were censored. The completion event list is not the inverse of the above drop-out event list, as there are quite a few entries where an individual did not meet criterion, but did not drop out, either. In these cases, the individual was shifted to a different task, which was not more advanced than the previous task. The results of this model are shown in Table S3. No personality dimensions were associated with survival time and completion of different stages.

Second, we excluded stages where the chimpanzee did not reach criterion, and fitted a Poisson GLMM to the number of trials it took an individual to reach criterion at a given stage. We again included random effects for individual and stage. The results of this model are shown in Table S3.

| **Table S3.** Models of learning speed from study 1 | | | |  |  |  |  |
| --- | --- | --- | --- | --- | --- | --- | --- |
|  | Cox model of completed stages | | |  | Poisson model of trials to criterion | | |
| Parameter | *β* | 95% | C.I. |  | *β* | 95% | C.I. |
| Dominance | -0.21 | [-1.78, | 1.36] |  | 0.74 | [-0.29, | 1.76] |
| Conscientiousness | -0.13 | [-1.20, | 0.94] |  | **0.88** | **[0.03,** | **1.74]** |
| Openness | 0.02 | [-1.43, | 1.47] |  | 0.96 | [-0.66, | 0.85] |
| Neuroticism | 0.29 | [-1.48, | 2.07] |  | -0.15 | [-1.44, | 1.14] |
| Agreeableness | 0.49 | [-0.62, | 1.59] |  | -0.57 | [-1.22, | 0.08] |
| Extraversion | -0.28 | [-1.41, | 0.85] |  | -0.29 | [-0.97, | 0.39] |
|  |  |  |  |  |  |  |  |

The significant effect of Conscientiousness on number of trials to reach criterion is likely due to the bias of high Conscientiousness individuals staying in the study for longer. Individuals who remained in the study were exposed to more and more difficult stages of the task, which would take more and more trials to complete. The power to detect the effect of Conscientiousness in this Cox model was 73%

*Accuracy*

We included all training and testing trials in our analyses of accuracy. Across all GLMMs we included random effects of participant, stage, and trial type (training or probe, *cf.* Sonnweber at al. [5]).

Our initial model included only personality, predicting correctness of response as a binomial outcome (Table S4). We also wished to establish whether or not there were training effects, so we updated our model by adding date as an additional covariate. Including date significantly improved model fit (likelihood-ratio test; *χ^2^* = 56.4, df = 1, *p* < 0.0001). To test for within-session effects, we added the trial number to the model, but this did not improve fit (likelihood-ratio test; *χ^2^* = 0.042, df = 1, *p* ≈ 0.84). This left the model which included personality and date as the best fit for the data (Table 1, main text).

| **Table S4.** Initial GLMM of accuracy in study 1 | | | |  |
| --- | --- | --- | --- | --- |
| Parameter | *β* | 95% | CI |  |
| Dominance | **-0.25** | **[-0.51,** | **0.00]** |  |
| Conscientiousness | **0.28** | **[0.04,** | **0.53]** |  |
| Openness | 0.11 | [-0.15, | 0.37] |  |
| Neuroticism | -0.04 | [-0.37, | 0.28] |  |
| Agreeableness | -0.18 | [-0.38, | 0.03] |  |
| Extraversion | **0.27** | **[0.07,** | **0.46]** |  |
|  |  |  |  |  |

The power to detect the effects of Dominance, Conscientiousness, and Extraversion was calculated for these three models of accuracy. In the initial model (Table S4), power was 81%, 93%, and 96%, respectively. In the model in including date (Table 1), power was 63%, 65%, and 88%, respectively. In the model including date and trial (Table S4), power was 59%, 67%, and 83%, respectively.

*Response Time*

The outcome variable, response time (RT), was log transformed for use in all regressions. We fit two sets of models: the first modelled all RT data, the second modelled only RT data from trials where the participant responded correctly, i.e. error free trials. Our models of the latter data were used in our interpretations of RT, though they did not markedly differ from the models which included all trials.

Our initial model of all log transformed RTs only included personality (Table S5). Including date did not improve the model fit (likelihood-ratio test; *χ^2^* = 3.0, df = 1, *p* ≈ 0.08). Our initial model of the log transformed RTs from correct responses again included only personality (Table S5), and again, adding date did not significantly improve the fit of the model (likelihood-ratio test; *χ^2^* = 0.2, df = 1, *p* ≈ 0.65).

| **Table S5.** Log-gamma GLMMs of RT data in study 1 | | | | |  |  |  |
| --- | --- | --- | --- | --- | --- | --- | --- |
|  | All trials | |  |  | Correct trials | | |
| Parameter | *β* | 95% | CI |  | *β* | 95% | CI |
| Dominance | 0.14 | [-0.46, | 0.74] |  | 0.10 | [-0.42, | 0.61] |
| Conscientiousness | **-0.61** | **[-1.05,** | **-0.16]** |  | **-0.53** | **[-0.92,** | **-0.14]** |
| Openness | **-0.51** | **[-0.94,** | **-0.08]** |  | **-0.38** | **[-0.76,** | **-0.01]** |
| Neuroticism | 0.09 | [-0.63, | 0.82] |  | -0.03 | [-0.69, | 0.62] |
| Agreeableness | 0.40 | [-0.06, | 0.75] |  | 0.27 | [-0.05, | 0.60] |
| Extraversion | **-0.47** | **[-0.79,** | **-0.15]** |  | **-0.39** | **[-0.67,** | **-0.10]** |
|  |  |  |  |  |  |  |  |

*Touch patterns*

The experimental task recorded the number of touches an individual made to the screen on every trial. While some individuals were quite precise, some were sloppier than others and sometimes simple errors occurred.

We fit GLMMs using a Poisson link function, as the data were counts. Our initial model included only personality variables. Including date as a covariate did not significantly improve model fit (likelihood-ratio test; *χ^2^* = 3.6, df = 1, *p* ≈ 0.06), so our final and initial models were the same (Table S7). High Conscientiousness chimpanzees tended to make fewer touches to the screen on any given trial. The power to detect this effect was 77%.

| **Table S6.** Poisson model of number of touches, per trial | | | |
| --- | --- | --- | --- |
| Parameter | *β* | 95% | CI |
| Dominance | -0.09 | [-0.54, | 0.34] |
| Conscientiousness | **-0.38** | **[-0.76,** | **-0.00]** |
| Openness | -0.15 | [-0.49, | 0.19] |
| Neuroticism | -0.01 | [-0.57, | 0.54] |
| Agreeableness | 0.13 | [-0.16, | 0.42] |
| Extraversion | -0.09 | [-0.39, | 0.21] |
|  |  |  |  |
|  |  |  |  |
|  |  |  |  |

***Study 2***

*Engagement*

Engagement data were based on 20 days when testing occurred in the outdoor research area, and 28 days which took place in the indoor research pods. Engagement data was collected for every individual, for every day of testing. An individual was assigned to one of three escalating levels: 0 – the individual did not enter the research area or did not show any interest in the touchscreen, 1 – the individual showed interest in and approached the touchscreen, but did not complete any trials, and 2 – the individual interacted with the touchscreen and completed as least one trial.

While the individuals who never participated and showed no interest tended not to waver in this behaviour, most individuals who showed interest and approached the screen but did not complete a trial did at some point participate in multiple trials of the task. In order to visualize trends in chimpanzees’ behaviour across all sessions, we plotted all six personality dimensions, split into three groups: those who never participated, those who completed multiple trials, and those that completed entire sessions (Figure S3).


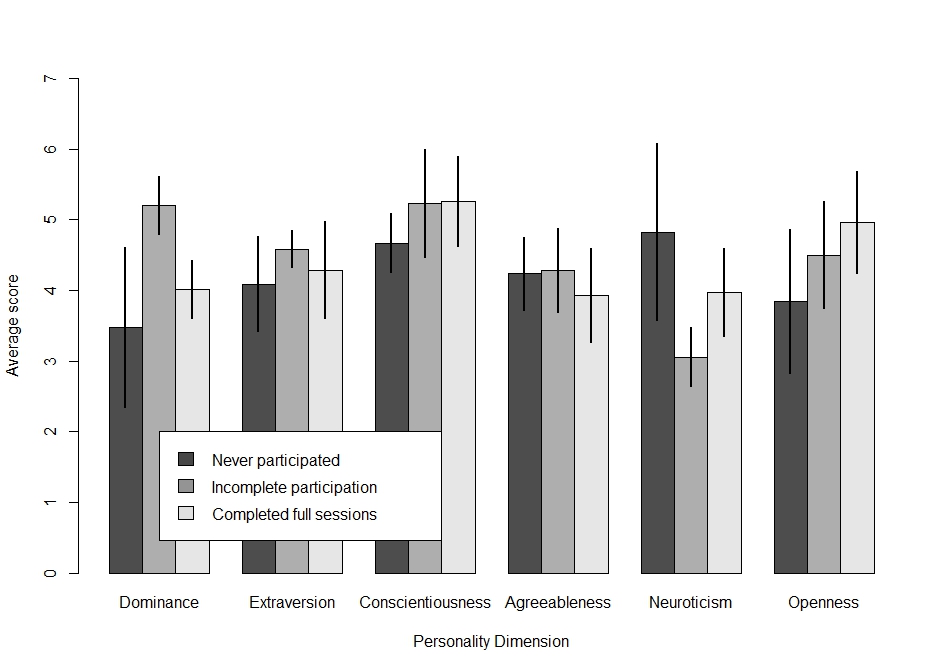


**Figure S3.** Personality of chimpanzees, split by level of participation in Study 2.

*Note.* Error bars represent standard errors around the mean.

To account for the ordered categorical nature of the engagement data, we fit cumulative link mixed models (CLMM) to assess how personality predicted participation. In all models, participant was included as a random effect, and location (indoor or outdoor research area) was included as a fixed effect for technical reasons.

Our base model included only personality and location as predictors. Because of what appeared to be non-linear relationships between participation and both Dominance and Neuroticism (Figure S3), we added quadratic effects for both predictors. Neither quadratic Dominance (likelihood-ratio test; *χ^2^* = 1.4, df = 1, *p* ≈ 0.24), nor quadratic Neuroticism (likelihood-ratio test; *χ^2^* = 0.2, df = 1, *p* ≈ 0.65) improved fit, so the final model only included linear personality predictors (Table 2, main text).

*Accuracy*

Performance analyses were based on a total of 1870 trials from 14 chimpanzees. As in study 1, our first model included only personality predictors, and we fit a second model which included date as an additional covariate, to probe for training effects. Including date did not significantly improve the fit of the model (likelihood-ratio test; *χ^2^* = 1.6, df = 1, *p* ≈ 0.20). Our initial and final models were thus the same (Table 2, main text). The power to detect the effect of Openness in this model was 50%.

*Response Time*

Two RT periods were available for analysis: the time between presentation of the sample screen and response, and the time between presentation of the test screen and response. We labelled the first of these “processing time” (PT) and the second “inspection time” (IT). PT and IT were both log-transformed before being regressed.

As in previous models, we entered date as an additional predictor and evaluated its effect on fit, and found a significant improvement (likelihood-ratio tests; PT: *χ^2^* = 287.6, df = 1, *p* < 0.0001; IT: *χ^2^* = 127.8, df = 1, *p* < 0.0001). We then entered trial to the model, which also significantly improved the IT model (*χ^2^* = 40.0, df = 1, *p* < 0.0001), but the PT model wold not converge when trial was included, so we settled on the PT model including date as our final model. In the final models (Table S7), date and trial showed significant, negative associations with RT measures, indicating that within and across training days, RTs became faster over time. We also found a significant relationship between Extraversion and both PT (power: 88%) and IT (power: 97%), indicating that higher Extraversion individuals responded faster.

| **Table S7.** RT models from study 2 | |  |  |  |  |  |  |
| --- | --- | --- | --- | --- | --- | --- | --- |
|  | Processing Time | |  |  | Inspection Time | |  |
| Parameter | *β* | 95% | CI |  | *β* | 95% | CI |
| Dominance | 0.44 | [-0.34, | 1.10] |  | 0.38 | [-0.02, | 0.77] |
| Conscientiousness | -0.20 | [-0.72, | 0.52] |  | -0.14 | [-0.44, | 0.15] |
| Openness | -0.22 | [-0.74, | 0.13] |  | -0.14 | [-0.43, | 0.15] |
| Neuroticism | -0.49 | [-1.52, | 0.53] |  | -0.41 | [-0.91, | 0.09] |
| Agreeableness | 0.02 | [-0.47, | 0.51] |  | 0.03 | [-0.23, | 0.30] |
| Extraversion | **-0.54** | **[-0.98,** | **-0.10]** |  | **-0.36** | **[-0.60,** | **-0.13]** |
| Date | -0.42 | [-0.47, | -0.37] |  | -0.42 | [-0.48, | -0.37] |
| Trial |  |  |  |  | -0.18 | [-0.22, | -0.14] |
|  |  |  |  |  |  |  |  |

We also analysed PT and IT including only correct trials. The models featuring date and trial were the best fit (PT: *χ^2^* = 133.8, df = 2, *p* < 0.0001; IT: 167.8, df = 2, *p* < 0.0001). The final models are shown in Table S8; the models differed from those which analysed all trials: faster PT was associated with lower Dominance, and higher Extraversion (power: 95%, 84%), while faster IT was associated with lower Dominance, and higher Extraversion, Agreeableness, and Neuroticism (power: 77%, 86%, 26%, 77%). Date and trial continued to have similar effects. As in study 1, we used the final models of the correct trials for interpretation.

| **Table S8.** RT models of correct responses from study 2 | | | | |  |  |  |
| --- | --- | --- | --- | --- | --- | --- | --- |
|  | Processing Time | |  |  | Inspection Time | |  |
| Parameter | *β* | 95% | CI |  | *β* | 95% | CI |
| Dominance | **0.54** | **[0.07,** | **1.01]** |  | **0.49** | **[0.24,** | **0.74]** |
| Conscientiousness | -0.13 | [-0.47, | 0.20] |  | -0.01 | [-0.22, | 0.19] |
| Openness | -0.18 | [-0.16, | 0.53] |  | -0.11 | [-0.10, | 0.31] |
| Neuroticism | -0.49 | [-1.09, | 0.09] |  | **-0.49** | **[-0.77,** | **-0.20]** |
| Agreeableness | -0.03 | [-0.34, | 0.28] |  | **-0.25** | **[-0.44,** | **-0.06]** |
| Extraversion | **-0.34** | **[-0.63,** | **-0.05]** |  | **-0.39** | **[-0.55,** | **-0.23]** |
| Date | -0.38 | [-0.45, | -0.30] |  | -0.39 | [-0.47, | -0.32] |
| Trial | -0.12 | [-0.18, | -0.06] |  | -0.18 | [-0.24, | -0.13] |
|  |  |  |  |  |  |  |  |

***Study 3***

*Progression of training*

The first four phases presented combinations of horizontal buttons. The buttons were randomly placed on the screen, and each was associated with a category of sound: pop music, classical music, or silence. The buttons are shown in representative presentations in Figure S4.

a)
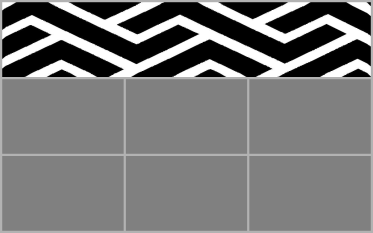
 b)
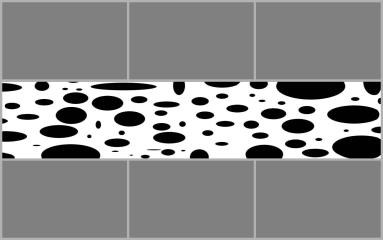
 c)
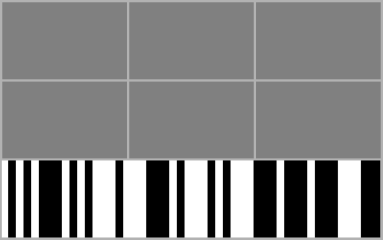


**Figure S4**. Images of the three touchscreen buttons, as they appeared during phases 1 - 4. When pressed, each initiated the following actions: (a) turned on classical music for three seconds, (b) turned music off / continued silence for three seconds and (c) turned on pop music for three seconds.

After pressing a button and receiving a reward 10 times, an individual would progress to the next phase. If an individual did not complete a phase within a single approach to the touchscreen, then the remaining button presses were completed the next time the individual approached the touchscreen, whether it was later in the session or on another day.

In phase 5, the task changed slightly, and is visually described in Figure S5.


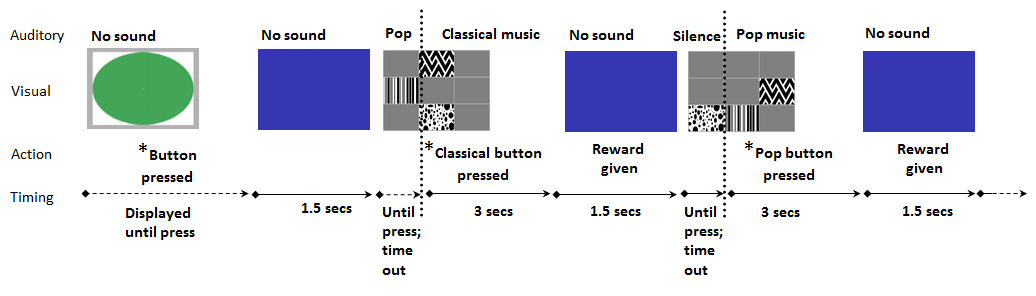


**Figure S5**. Example first two trials during a session of phase 5, progressing from left to right. Phase 5 continued until 40 buttons, not including the green start button, had been successfully pressed. If the touchscreen was not interacted with for 30 seconds, it reverted back to the green circle screen.

Individuals had to complete 40 trials; 10 where the appearance of the buttons on the screen coincided with classical music playing at onset of the grid screen, 10 in which buttons appeared with pop music playing, and 20 where no music accompanied the appearance of the grid screen. The order of these trials was randomised. Again, if an individual did not complete the testing within a single approach of the touchscreen or experimental session then the remaining button presses were completed the next time the individual approached the touchscreen, whether it was later in the session or on another day.

In phase 6, rewards were no longer given out for pressing buttons on the touchscreen. To encourage the chimpanzees to enter the research pods, a bale of straw (approximately 10kg) and 7kg of primate pellets were spread across the two pods. As the chimpanzees were let into the research pods the touchscreen displayed the three buttons in a randomised positions on the 3x3 grid. For three sessions classical music was already playing as the individuals entered the pods, for three sessions pop music was playing, for three sessions there was silence, and for three sessions the touchscreen was not physically available to the participants and no music played (total of 12 sessions). The sound would continue until a button was pressed or the trial ended after 60 minutes. If an individual approached the touchscreen and pressed a button, the corresponding genre of music would play or the music would be turned off until a new button was pressed. If the touchscreen was silent and the silence button was pressed then silence would continue. If music was playing and the same music button was pressed, a different randomly selected piece of music from the same category would begin playing. If no new button was pressed that music or silence would continue until the end of the trial. Otherwise, the task procedure was the same as in Figure S5. Data was collected on how long individuals were present in the pod, how many approaches were made to the screen, and how long individuals spent in front of the screen.

*Differences in trained vs. untrained groups*

As in study 1, chimpanzees were split into two groups, participants and non-participants, and personality was plotted having been divided along these lines (Figure S6).
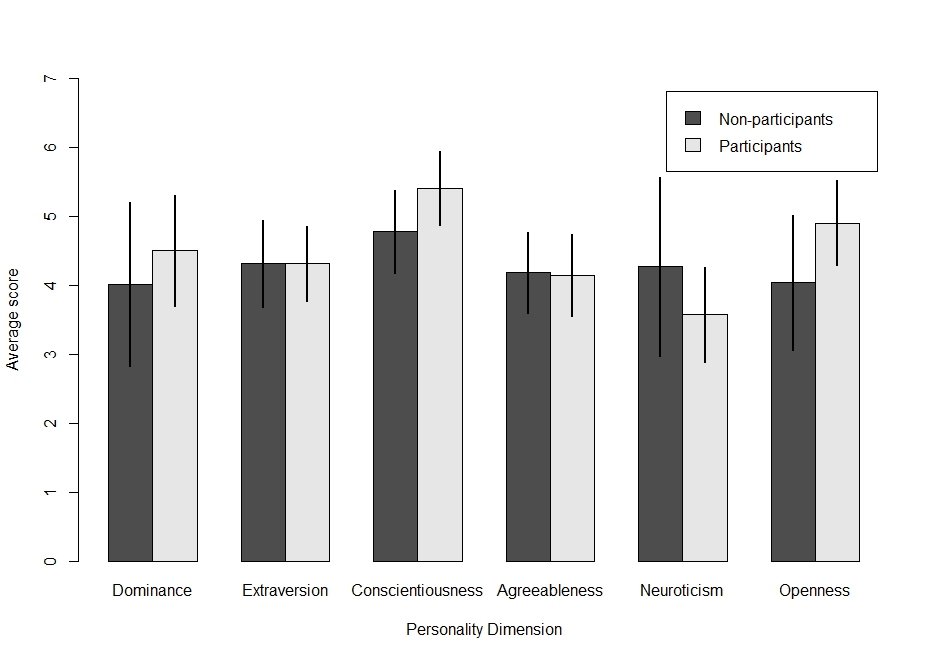


**Figure S6.** Personality of chimpanzees, split by level of participation in Study 3. Error bars represent standard errors around the mean.

*Time spent in the research pods*

The time data were inflated with zeroes as some chimpanzees never entered the pods on certain days. Zero-inflation is difficult to model with GLMMs, though the presence of zeroes does not necessarily mean that the assumption of the Poisson model are violated [7].

Thus, we first fit a Poisson GLMM to the model, and tested for overdispersion. This model was overdispersed (*χ^2^* = 2827, df = 207, *p* < 0.0001), so we modelled these data using a negative binomial (NB) GLMM, which is often better suited for overdispersed data [8]. Moreover, the NB model fit the data better than the Poisson model (AIC_NB_ = 1519.4, AIC_Poisson_ = 3782.8), so we interpreted the output of the NB model (Table S9). The power to detect the effect of Extraversion in this model was 81%.

| **Table S9.** NB model of time spent in research pods | | | |
| --- | --- | --- | --- |
| Parameter | *β* | 95% | CI |
| Dominance | -0.14 | [-1.60, | 1.31] |
| Conscientiousness | -0.25 | [-1.13, | 0.63] |
| Openness | -0.06 | [-0.79, | 0.66] |
| Neuroticism | -0.61 | [-2.08, | 0.85] |
| Agreeableness | -0.28 | [-0.96, | 0.42] |
| Extraversion | **0.78** | **[0.06,** | **1.51]** |
|  |  |  |  |

*Approaches to the screen*

As the data included one line per chimpanzee, and some chimpanzees never approached the screen, the data had the potential to be zero-inflated. We first fit a Poisson model and tested for overdispersion, and concluded that the Poisson model was overdispersed (*z* = 2.71, *p* < 0.005). We again fell back on a NB model (Table S10), which fit the data better than the Poisson model (AIC_NB_ = 91.89, AIC_Poisson_ = 108.0).

| **Table S10.** NB model of number of approaches to screen | | | |
| --- | --- | --- | --- |
| Parameter | *β* | 95% | CI |
| Dominance | -0.19 | [-1.67, | 1.15] |
| Conscientiousness | **1.09** | **[0.15,** | **2.16]** |
| Openness | 0.46 | [-0.43, | 1.46] |
| Neuroticism | 0.10 | [-1.43, | 1.48] |
| Agreeableness | **-0.93** | **[-1.63,** | **-0.28]** |
| Extraversion | 0.69 | [-0.19, | 1.70] |
|  |  |  |  |

*Time spent at the screen*

Every time a chimpanzee approached the experimental apparatus, we timed how long the individual spent in front of and interacting with the touchscreen. Since the time spent at the screen was always at least a second, there were no zeroes in the data, and no need to accommodate inflation. 1 outlier greater than 3 standard deviations from the mean was removed. A Poisson GLMM was fit with personality covariates (Table S11). The power to detect the effects of Openness and Extraversion was 96% and 87%.

| **Table S11.** Poisson model of time spent at screen | | | |  |
| --- | --- | --- | --- | --- |
| Parameter | *β* | 95% | CI |  |
| Dominance | 0.26 | [-0.20, | 0.73] |  |
| Conscientiousness | 0.21 | [-0.17, | 0.59] |  |
| Openness | **0.52** | **[0.21,** | **0.83]** |  |
| Neuroticism | 0.41 | [-0.10, | 0.91] |  |
| Agreeableness | 0.19 | [-0.04, | 0.43] |  |
| Extraversion | **-0.36** | **[-0.66,** | **-0.05]** |  |
|  |  |  |  |  |

***References***

1. Herrelko, E. S., Vick, S.-J. & Buchanan-Smith, H. M. 2012 Cognitive research in zoo-housed chimpanzees: influence of personality and impact on welfare. *Am. J. Primatol.* **74**, 828–40. (doi:10.1002/ajp.22036)
2. Weiss, A., Inoue-Murayama, M., Hong, K.-W., Inoue, E., Udono, T., Ochiai, T., Matsuzawa, T., Hirata, S. & King, J. E. 2009 Assessing chimpanzee personality and subjective well-being in Japan. *Am. J. Primatol.* **71**, 283–92. (doi:10.1002/ajp.20649)
3. Shrout PE, Fleiss JL. 1979 Intraclass correlations: uses in assessing rater reliability. *Psychological bulletin.* **86**(2):420.
4. Tomonaga, M. & Imura, T. 2015 Efficient search for a face by chimpanzees (Pan troglodytes). *Sci. Rep.* **5**, 11437. (doi:10.1038/srep11437)
5. Sonnweber, R., Ravignani, A. & Fitch, W. T. 2015 Non-adjacent visual dependency learning in chimpanzees. *Anim. Cogn.* (doi:10.1007/s10071-015-0840-x)
6. Gelman A. Scaling regression inputs by dividing by two standard deviations. *Statistics in medicine.* 2008 Jul 10;27(15):2865-73.
7. Warton, D. I. 2005 Many zeros does not mean zero inflation: Comparing the goodness-of-fit of parametric models to multivariate abundance data. *Environmetrics* **16**, 275–289. (doi:10.1002/env.702)
8. Lindén, A. & Mäntyniemi, S. 2011 Using the negative binomial distribution to model overdispersion in ecological count data. *Ecology* **92**, 1414–1421. (doi:10.1890/10-1831.1)
